# Comparative Study on the Outcomes of Right Ventricular Outflow Tract Stenting *vs.* Modified Blalock-Taussig Shunt in Patients with Tetralogy of Fallot: A Prospective Randomized Trial

**DOI:** 10.21470/1678-9741-2023-0478

**Published:** 2025-03-17

**Authors:** Aleksey V. Voitov, Meline G. Morsina, Serezha N. Manukian, Ilya A. Soynov, Nataliya R. Nichay, Yury Yu. Kulyabin, Aleksey N. Arkhipov, Manolis G. Pursanov, Artem V. Gorbatykh, Alexander V. Bogachev-Prokophiev

**Affiliations:** 1 Meshalkin National Medical Research Center, Ministry of Health of Russian Federation, Novosibirsk, Russian Federation; 2 Health Department, Morozov Children's Municipal Clinical Hospital of the Moscow City, Moscow, Russian Federation; 3 Almazov National Medical Research Centre, St. Petersburg, Russian Federation

**Keywords:** Tetralogy of Fallot, Pulmonary Artery, Blalock-Taussig Procedure, Stents, Sepsis

## Abstract

**Objective:**

To evaluate pulmonary vascular development and outcomes of complete correction following palliative treatment in infants with critical tetralogy of Fallot.

**Methods:**

This prospective, randomized, two-center study included infants with tetralogy of Fallot who underwent surgery between June 2018 and 2022. The patients were divided into two groups - those who underwent stenting of the right ventricular outflow tract (stent group, n=21) and those who underwent modified Blalock-Taussig shunt placement (shunt group, n=21).

**Results:**

In the stent group, a significantly greater increase in Nakata index was observed, with mean values rising from 104.2 to 208.6 mm^2^/m^2^, compared to an increase from 107.3 to 169.4 mm^2^/m^2^ in the shunt group (P<0.01). According to the mixed model analysis, the rate of growth of the right pulmonary artery in the stent group was 2.05*10-2 z score/day, which was 3.01 times greater than that in the shunt group (P<0.01). The rate of growth of the left pulmonary artery in the stent group was 2.3*10-2 z score/day, which was 1.47 times greater than that in the shunt group (P<0.01). In one patient (4.8%), after 76 days following the stenting of the RVOT, a severe infectious process with sepsis occurred, leading to a fatal outcome. Complete correction in the stent group involved transannular patch repair of the right ventricular outflow tract to the pulmonary artery in 12 patients (60%), while the same procedure was performed in 15 patients (71.4%) in the shunt group (P=0.52).

**Conclusion:**

Stenting of the right ventricular outflow tract provides hemodynamic stabilization and symmetric growth of the pulmonary vascular bed compared to the formation of a modified Blalock-Taussig shunt.

## INTRODUCTION

**Table:** 

Abbreviations, Acronyms & Symbols				
CI	= Confidence interval		PA	= Pulmonary artery
ICU	= Intensive care unit		PV	= Pulmonary valve
LPA	= Left pulmonary artery		RPA	= Right pulmonary artery
LVEF	= Left ventricular ejection fraction		RVOT	= Right ventricular outflow tract
mBTs	= Modified Blalock-Taussig shunt		ToF	= Tetralogy of Fallot
MSCT	= Multislice computed tomography		VSD	= Ventricular septal defect
OR	= Odds ratio			

The management of symptomatic infants with tetralogy of Fallot (ToF) has undergone changes over the years. Temporary palliation remains a crucial option for managing cyanotic patients with low birth weight or complex anatomy in most centers, as complete repair in these cases carries significantly higher risks compared to repair at an older age^[1]^. Independent risk factors for complete neonatal repair and palliation with a modified Blalock-Taussig shunt (mBTs) include low birth weight and small pulmonary arteries (PAs)^[1,2]^. Limited reports have assessed pulmonary arterial growth after the mBTs procedure^[3-5]^. Several studies have indicated enhanced pulmonary arterial growth after the Norwood procedure in hypoplastic left heart syndrome, utilizing a right ventricle to PA conduit compared to an mBTs^[6,7]^. On the other hand, relieving the right ventricular outflow tract (RVOT) obstruction in patients with Fallot-type lesions through stent implantation may promote better pulmonary arterial growth compared to an mBTs. Surgical strategies such as modified mBTs or primary neonatal repair might lead to potential complications, such as pulmonary over-circulation or even PA stenosis^[8]^. These surgical strategies have been associated with relatively high mortality rates, reported as 6.2% and 7.8%, respectively^[9]^. An alternative approach to palliation, such as RVOT stenting, has emerged due to the accepted optimum timing for complete repair being between three and 11 months^[10]^. Previous studies on this technique have consistently reported favorable results, demonstrating excellent outcomes with a low incidence of complications. Several studies have demonstrated that in patients with hypoplastic left heart syndrome, pulmonary atresia, and ventricular septal defect (VSD) (types A and B according to Tchervenkov classification), reconstruction of the RVOT yields better outcomes in terms of PA rehabilitation compared to the formation of an mBTs^[11-14]^. Therefore, we expected that the resolution of significant obstruction in the RVOT in ToF may result in more efficient growth of the central PA compared to the utilization of the mBTs. The aim of our study was to compare the outcomes of two different palliative approaches, specifically RVOT stenting *vs.* modified mBTs, in the context of a two-stage correction of ToF.

## METHODS

**Fig. 1:**
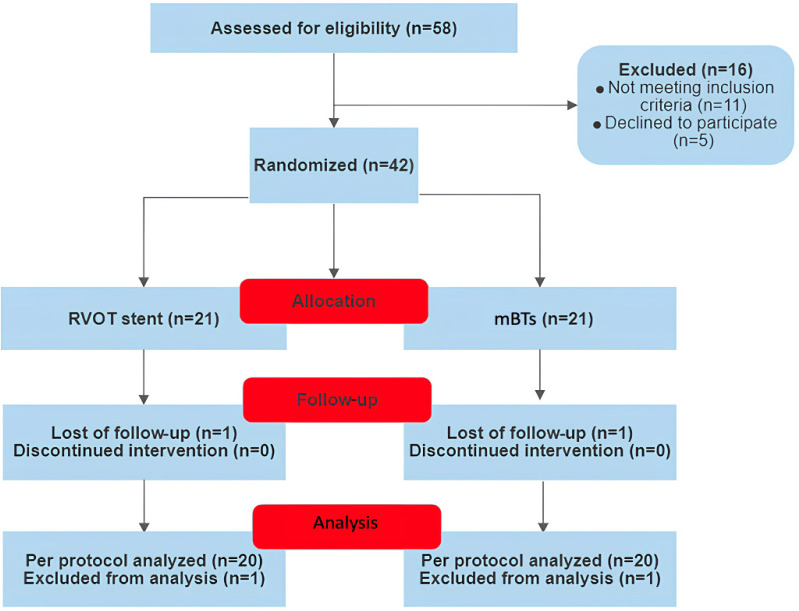
Patient enrolment flowchart. mBTs=modified Blalock-Taussig shunt; RVOT=right ventricular outflow tract.

Between June 2018 and August 2022, a total of 42 consecutive patients with symptomatic ToF were included in the study. This study followed a multicenter approach and utilized a prospective, open, and randomized design with two arms. The study was carried out in accordance with the Declaration of Helsinki and was approved by the institutional review board (Nº 9 from 17 May 2018). According to the inclusion and exclusion criteria (Figure 1), a total of 42 infants who underwent staged correction of the defect were included in the study out of the initial 58 patients. Informed consent was obtained from each patient (from their legal representatives). After randomization, the children were divided into two groups (21 patients in each group) based on the method of palliative treatment: either stenting of the RVOT or creation of an mBTs. The patients’ baseline characteristics are given in Table 1. Infants diagnosed with ToF, aged < 4 months, scheduled to undergo palliative surgery, and meeting the inclusion criteria of having confluent PA, a Nakata index of ≥ 150 mm^2^/m^2^, and a PA *z* score of < -2 were included in the study. Patients who had discordant atrioventricular and/or ventriculoarterial connections, contaminant cardiac pathologies including common atrioventricular canal (CAVC), multiple VSDs, or coronary anomalies, genetic pathologies, or those who had undergone other surgical approaches such as complete repair, central shunt placement, or patent ductus arteriosus stenting were excluded from the study. Around 55% of the patients underwent initial palliation due to either significant spells or severe desaturation, or the presence of hypoplastic PA with a *z* score of < -2. At the time of intervention, over 70% of the patients were receiving medical treatment, including the administration of beta-blockers or prostaglandin infusion. Complete repair beyond early infancy was postponed in cases where there were other relevant comorbidities, such as syndromes, prematurity, or low birth weight, and if the patient was clinically stable.

**Table 1:** Patients’ baseline data

	RVOT (n=21)	mBTs (n=21)	*P*-value
Male, *n* (%)	8 (38.1)	7 (33.3)	0.103
Age (days)	78 (18;116)	70 (24;118)	0.165
Weight (kg)	3.4 (2.85;4.55)	3.3 (2.68;4.25)	0.263
O_2_ saturation (%)	69 (60;78)	71 (69; 74)	0.298
Nakata index (mm^2^/m^2^)	121.17 (105.72;134.61)	135.81 (110.54;138.74)	0.448
McGoon ratio	1.09 (1.07;1.33)	1.29 (1.2; 1.36)	0.132
Reddy index	60.87 (51.05;70.7)	53.86 (46.8;60.9)	0.212
*z* score PA	-3.8 (-4.7;-2.8)	-3.9 (-4.3;-3.5)	0.822
*z* score RPA	-2.3 (-2.8;-1.7)	-2.3 (-3;-1.9)	0.269
*z* score LPA	-2.1 (-2.4;-1.3)	-1.9 (-2.5;-1.7)	0.562

LPA=left pulmonary artery; mBTs=modified Blalock-Taussig shunt; PA=pulmonary artery; RPA=right pulmonary artery; RVOT=right ventricular outflow tract

This study aims to compare the degree of development of the central PAs following palliative intervention, describe complications in the interstage period, and evaluate the outcomes of delayed anatomical correction. The primary endpoint was the degree of development of the central PAs at three months following the palliative operation. The effectiveness of the palliative treatment was considered achieved if the Nakata index was ≥ 200 mm^2^/m^2^ (evaluated using binary logistic regression).

The secondary endpoints included evaluating early and mid-term postoperative complications such as shunt thrombosis, bleeding, infective endocarditis, stent thrombosis, and reinterventions.

The growth dynamics of the pulmonary valve, main PA, and right and left branches of the PA at the level of bifurcation were evaluated using a series of echocardiographic examinations conducted prior to the palliative treatment, during the interstage period on an outpatient basis, and at the time of delayed anatomical correction. The results were analyzed using indexed *z* score values^[15]^. To assess the anatomy of the RVOT, degree of PA hypoplasia, and coronary artery anatomy prior to the palliative intervention and at three months post-intervention, contrast-enhanced multislice computed tomography (MSCT) was performed. The degree of PA hypoplasia was evaluated using the Nakata index, McGoon ratio, and Reddy index^[16,17]^.

### Surgical Techniques

#### Modified Blalock-Taussig Shunt Technique

All mBTs procedures were performed through a sternotomy. Following a complete sternotomy, the excision of the right lobe of the thymus was performed, and the course of the brachiocephalic trunk was dissected up to its bifurcation. Additionally, the dissection of the right PA was carried out up to the hilum. A bolus of 100 IU/kg crystalline heparin was administered. The brachiocephalic trunk was clamped with a Cooley clamp, and the distal right subclavian artery, temporarily with a ligaclip. A longitudinal arteriotomy was then made at the previously marked undersurface of the continuity between the truncus and subclavian artery. The thin-wall Gore-Tex stretch vascular graft (W. L. Gore & Associates, Inc., Arizona, United States of America) was sutured to the arteriotomy in an oblique fashion, with one end of the graft connected end-to-side. Clamps were released and a good shunt flow through the anastomosis ascertained. The Cooley clamp was placed again to exclude the proximal anastomosis from the circulation. The shunt length was trimmed so as to avoid it being too long. The right PA was excluded from circulation using two vascular clamps. The transversely fashioned distal end of the graft was anastomosed to the right PA.

#### Right Ventricular Outflow Tract Stenting Procedure

**Fig. 2:**
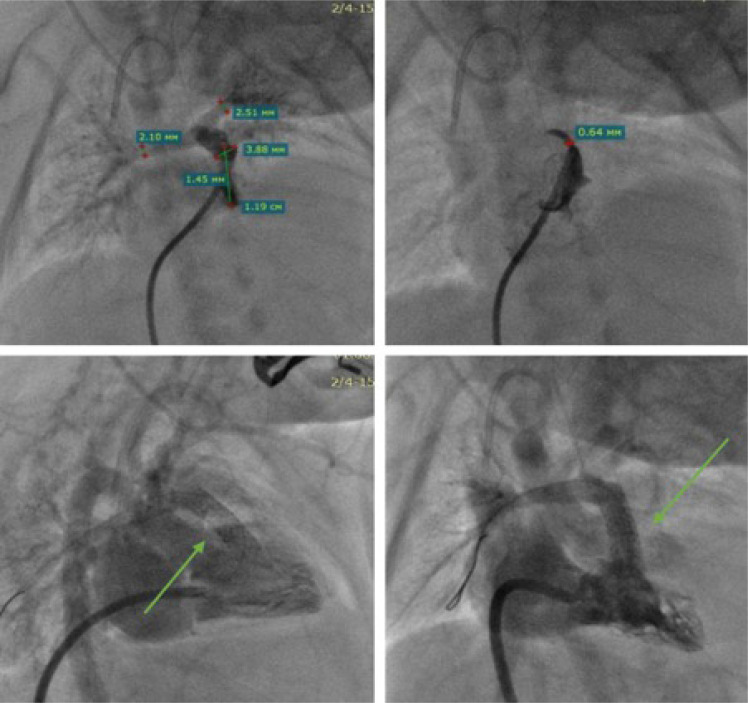
Right ventricular outflow tract stenting procedure, stent placed across the pulmonary valve achieving a two-point fixation, one at infundibular and another at valve annulus level.

**Fig. 3:**
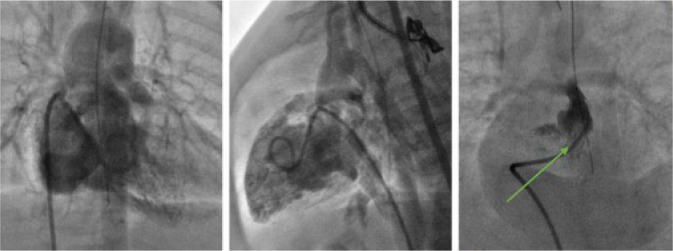
Right ventricular outflow tract stenting.

All cardiac catheterizations were performed under general anaesthesia with fluoroscopic guidance. Catheterization was done through the right or left femoral venous approach. The patients were given 50-100 IU/kg of heparin in two doses (50 IU/kg first and 50 IU/kg half hour later) to maintain an activated clotting time of > 200 s after catheterization. Prophylactic intravenous antibiotic with 30 mg/kg of cefazolin was administrated at the beginning of the procedure and in two subsequent doses every eight hours during the following 24 hours. The stenting procedure involved the use of bi-plane angiography and accessing the RVOT through the femoral veins. A right ventricular cineangiogram is performed through a pigtail or any other diagnostic catheter placed within the apex of the right ventricle. A telescopic configuration of catheters was used, along with a guide wire, to cross the RVOT. A coronary wire was securely positioned in the distal right/left PA. After stabilization of the guide wire, balloon angioplasty of the RVOT and the main PA was performed to alleviate the difficulties of stent implementation into the RVOT (Figure 2). The stents were selected based on the size of the infants, RVOT infundibular length, and anticipated length of palliation. After the selection of the stent, the appropriate delivery sheath or guide catheter is used. Peripheral stents with nominal diameters that were 1-2 mm greater than the annular dimension were selected and implanted in the RVOT. If the annulus is small or if there is supravalvular PA narrowing, the stent may be placed across the pulmonary valve achieving a two-point fixation, one at infundibular and another at valve annulus level. Given these anatomical features, longer stents, measuring 14-16 mm in length, were used in such cases. In instances where marked pulmonary valve hypoplasia was not observed, and future valve-sparing procedures were anticipated, stents were exclusively implanted in the RVOT. In these cases, the stent diameter was chosen to be 2-3 mm larger than the RVOT. The stents were 5-7 mm in diameter and 12-16 mm in length. The stent position is verified using the side arm of the sheath on the check angiogram (Figure 3). The final angiogram records the stent position, opacification of the branch PAs, and pulmonary valve movements. Before removing the catheter, echocardiography was performed to confirm the position of the stent, assess any impact on tricuspid valve function, and check for signs of effusion. The results indicated a significant increase in pulmonary blood flow and oxygen saturation.

#### Complete Correction

Complete correction surgery was performed in all cases under conditions of cardiopulmonary bypass, moderate hypothermia, and with the use of Custodiol® cardioplegic solution. The choice of the method for reconstructing the RVOT was based on the assessment of the risk of developing right ventricular insufficiency.

### Statistical Analysis

The sample size calculation was performed using G*Power 3.1 (http://gpower.hhu.de), considering an anticipated difference of 40% in mean deposition, with a statistical power of 80%. To account for potential incomplete observations during the follow-up period, the calculated sample size was increased by 10% to adequately compensate for any missing data. Continuous variables are expressed as median (25^th^; 75^th^ percentile), unless otherwise specified. Categorical variables are presented as numbers (%). The Mann-Whitney U test, chi-square test, and Fisher’s exact test were used for intergroup comparisons. Multiple logistic regression analysis was performed to detect risk factors for the occurrence of complications (residual shunts). The multivariate model considered the significant variables (*P*<0.05) in univariate analysis, and odds ratios and their 95% confidence intervals were calculated. *P*<0.05 was considered statistically significant. Statistical analysis was performed by using Stata 14 (StataCorp LP, College Station, Texas, United States of America) and MS Excel 2022 software (Microsoft Corporation, Redmond, Washington, United States of America).

## RESULTS

### Pulmonary Development

**Fig. 4:**
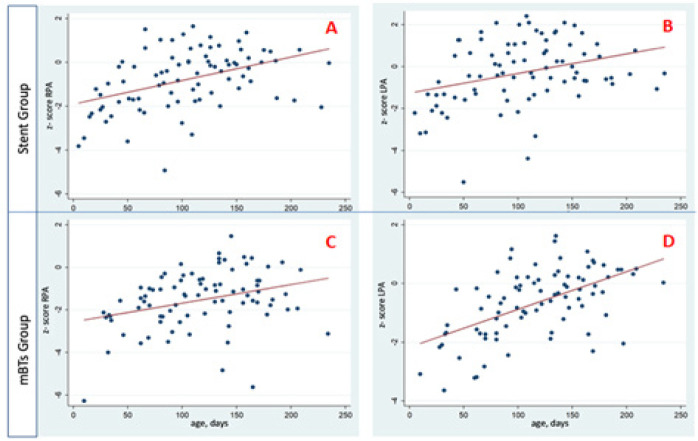
Development of pulmonary artery branches (by z score) during the observation period in the stent and shunt groups. LPA=left pulmonary artery; mBTs=modified Blalock-Taussig shunt; RPA=right pulmonary artery. A, B=development of pulmonary artery branches in the stent group; C, D=development of pulmonary artery branches in the shunt group. The red line represents the predicted growth trajectory of the pulmonary artery.

A comparative assessment of the growth dynamics of the central PAs was conducted from the time of palliative intervention to delayed anatomical correction using indexed parameters. In the intergroup comparison, there were no significant differences in the degree of development of the central PAs between the stent and mBTs groups prior to palliative treatment (*z* score growth 2.2 in stent group [*P*<0.01], *z* score growth 1.3 in mBTs group [*P*<0.01]). By the time of complete correction, no significant growth of the pulmonary valve was observed in the study groups (*P*=0.5 in the stent group, *P*=0.1 in the mBTs group). However, there was noted development of the branches of the PA. During the observation period, the median *z* score of the right PA increased from -2.3 to 0.08 in the stent group (*P*<0.01), and from -2.3 to -1.5 in the mBTS group (*P*<0.01), respectively. The median *z* score of the left PA in the stent group increased from -2.1 to 0.5 (*P*<0.01), while in the mBTs group, it increased from -1.9 to -0.25 (*P*<0.01). Figure 4 displays all the measurements of the PA branches based on echocardiography data. The growth dynamics of the PAs in terms of *z* scores during the observation period are presented in Table 2 and Figure 4. According to contrast-enhanced MSCT data obtained three months after palliative treatment, there was also noted growth of the PA based on the Nakata index. In the stent group, the mean Nakata index increased from 121.17 mm^2^/m^2^ to 208.6 mm^2^/m^2^ (*P*<0.01), and in the mBTs group, it increased from 135.81 mm^2^/m^2^ to 169.4 mm^2^/m^2^ (*P*<0.01). By the time of complete correction, in the intergroup comparison, the z scores of the right and left PAs, as well as the Nakata index, were significantly higher in the stent group compared to the mBTs group (*P*<0.01, *P*=0.049, and *P*<0.01, respectively).

**Table 2:** Intergroup comparison of the development of the pulmonary pathway from the time of palliative treatment to complete correction.

Characteristic	Stent group		*P*-value	mBTs group		*P*-value
	Before palliative (n=21)	Complete correction (n=20)		Before palliative (n=21)	Complete correction (n=21)	
*z* score PV	-3.3 (-4.5;-2.8)	-3.2 (-4.1;-2.6)	0.5	-3.8 (-5.7;-2.7)	-3.1 (-4.5;-2.4)	0.1
*z* score main PA	-3.8 (-4.7;-2.8)	-1.6 (-2.4;-0.8)	< 0.01	-3.9 (-4.3;-3.5)	-2.6 (-3.7;-1.9)	0.01
*z* score RPA	-2.3 (-2.8;-1.7)	0.08 (-0.4;0.9)	< 0.01	-2.3 (-3;-1.9)	-1.5 (-2.1;-0.6)	< 0.01
*z* score LPA	-2.1 (-2.4;-1.3)	0.5 (-0.4;1.7)	< 0.01	-1.9 (-2.5;-1.7)	-0.25 (-0.53;0.47)	< 0.01
Nakata index	121.17 (105.72;134.61)	208.6 (173;266)	< 0.01	135.81 (110.54;138.74)	169.4 (139.7;187.1)	< 0.01

LPA=left pulmonary artery; mBTs=modified Blalock-Taussig shunt; PA=pulmonary artery; PV=pulmonary valve; RPA=right pulmonary artery

To assess and compare the growth rate of the central PAs in the study groups, a mixed-effects model analysis was performed (Table 3). As a result, it was found that stenting of the pulmonary valve leads to a growth rate of the right PA at 2.05*10-2 *z* score per day (*P*<0.01), which is 3.01 times higher than the growth rate of 0.68*10-2 *z* score per day in the mBTs group. The growth rate of the left PA in the stent group was 2.23*10-2 *z* score per day, which is 1.47 times higher than the growth rate of 1.56*10-2 *z* score per day in the mBTs group (*P*-value < 0.01). None of the independent predictors (prematurity, neonatal status, weight before palliative treatment) significantly influenced the growth rate of the PAs.

**Table 3:** A mixed-effects model was used to compare the growth rate of the central pulmonary pathway in the study groups.

	Main PA	*P*-value	RPA	*P*-value	LPA	*P*-value
Stent group (*z* score/per day)	2.15^*^10^-2^	< 0.01	2.05^*^10^-2^	< 0.01	2.3^*^10^-2^	< 0.01
mBTs group (*z* score/per day)	1.23^*^10^-2^	< 0.01	0.68^*^10^-2^	0.02	1.56^*^10^-2^	< 0.01
Ratio of the growth rate of the PA in the stent group *vs.* the mBTs group	1.75		3.01		1.47	
Prematurity	-0.337	0.49	0.17	0.68	0.22	0.5
Newborn	0.046	0.9	-0.27	0.5	-0.16	0.6

LPA=left pulmonary artery; mBTs=modified Blalock-Taussig shunt; PA=pulmonary artery; RPA=right pulmonary artery

In order to determine the factors influencing the achievement of a Nakata index ≥ 200 mm^2^/m^2^ in the study groups, binary logistic regression was performed. The results are presented in Table 5. As a result, it was found that stenting of the RVOT increases the odds of achieving a Nakata index ≥ 200 mm^2^/m^2^ by 7.78 times compared to the formation of an mBTs.

**Table 5:** General characteristics of patients before radical correction.

Characteristics	Stent group	mBTs group	*P*-value
Age, days	151 (136;192.5)	182 (166;200)	0.081
Weight	6.1 (5.67;6.91)	6.2 (5.7;6.6)	0.984
SatO_2_, %	89.5 (86;93)	83 (78;85)	0.035
*z* score PV	-3.2 (-4.1;-2.6)	-3.1 (-4.5;-2.4)	0.702
*z* score main PA	-1.6 (-2.4;-0.8)	-2.6 (-3.7;-1.9)	0.029
*z* score RPA	0.08 (-0.4;0.9)	-1.5 (-2.1;-0.6)	0.022
∆*z* score RPA	2.2 (1.7;3.5)	1.1 (0.8;1.97)	0.018
*z* score LPA	0.5 (-0.4;1.7)	-0.3 (-0.5;0.5)	0.049
∆*z* score LPA	2.6 (1.7;3.6)	1.6 (1.1;2.4)	0.026
Nakata index, mm^2^/m^2^	208.6 (173;266)	169.4 (139.7;187.1)	0.014
∆ Mean Nakata index, mm^2^/m^2^	107.9 (84.5;131.9)	66.1 (48.8;83.4)	0.005
Mean index of growth (mm^2^/m^2^/month)	35.9 (28.1;43.8)	22.1 (16.3;27.8)	0.006
McGoon ratio	1.89 (1.75; 2.03)	1.57 (1.41; 1.73)	0.002
Reddy index (before complete repair)	108.48 (93.01;123.97)	99.17 (89.21;109.14)	0.292

LPA=left pulmonary artery; mBTs=modified Blalock-Taussig shunt; PA=pulmonary artery; PV=pulmonary valve; RPA=right pulmonary artery

Binary logistic regression was performed to assess the factors influencing the achievement of a Nakata index ≥ 200 mm^2^/m^2^ by the time of delayed anatomical correction.

### Interstage Period

In one patient (4.8%) after 76 days following the stenting of the RVOT, a severe infectious process with sepsis occurred, leading to a fatal outcome. No other complications requiring hospitalization during the interstage period or repeat interventions were observed in the remaining cases.

### Complete Correction

Delayed complete correction was performed on 20 patients in the stent group with an average of 97 (92;118) days after palliative intervention and on 21 infants in the mBTs group after 109 (106;128) days. General characteristics of the patients before the surgical treatment are presented in Table 5.

In patients of the stent group, the stent was located throughout the RVOT with ingrowth of the structures of the bare metal stent into the hypertrophied septoparietal trabeculae of RVOT. In 12 out of 20 children (55%), the stent crossed the pulmonary valve, while in the remaining nine cases (45%), it was isolated in the RVOT. In both groups, after palliative treatment, pulmonary valve hypoplasia was preserved with a median *z* score of -3.2 in the stent group and a median *z* score of -3.1 in the mBTs group; the leaflets of the valve were mostly represented by fibrotically altered cusps. Due to the persistent marked hypoplasia, the native valve was transected in all cases during complete repair. Intraoperatively, during the revision, the previously implanted stents were not deformed, not patent, and not covered with fibrous tissue around their perimeter. During the process of explantation, the stent was gradually dissected from the RVOT and extracted from the PA trunk without significant technical problems. In cases where the stent was intra-annularly positioned, the fibrotic leaflets of the pulmonary valve were resected simultaneously. Transannular plasties of the RVOT were performed in 12 (60%) out of 20 patients in the stent group and in 15 (71.4%) out of 21 children in the mBTs group (*P*=0.52). Valve-sparing reconstruction of the PA outflow with the Contegra™ No. 12 xenograft conduit (Medtronic, United States of America) was performed in eight children (40%) in the stent group and in six (28.6%) in the mBTs group (*P*=0.52). Due to the deformation and stenosis of the PA at the site of the previously formed shunt, the reconstruction of the PA outflow was complemented by the plasties of the stenosed branch.

In the mBTs group, the duration of the operation was significantly longer (*P*=0.046) compared to the stent group, which was due to the adhesive process in the pericardial cavity (Table 6). However, considering the additional time spent on explanting the stent from the RVOT, the duration of aortic occlusion and the duration of cardiopulmonary bypass were significantly longer in the stent group (*P*<0.01 and *P*<0.01, respectively). Due to the development of severe right ventricular insufficiency in the early postoperative period, one patient in the stent group (5%) and another patient in the mBTs group (4.8%) after transannular plasty required the establishment of extracorporeal membrane oxygenation (for 10 days in the stent group and for five days in the mBTs group), with subsequent favorable outcomes. Additionally, one case of stent placement in the branches of the PAs after prosthetics of the PA was registered in each of the studied groups. In the mBTs group, diaphragm plication was required in one case (4.8%) on the eighth day after the operation. No fatalities were reported during the hospitalization period (*P*>0.99).

**Table 6:** Intraoperative and postoperative parameters.

Characteristics	Stent group (n=21)	mBTs group (n=20)	*P*-value
Aortic occlusion, hours	71 (64;85)	41 (30;48)	< 0.01
Duration of artificial circulation, hours	104 (96;130,5)	77 (63;85)	< 0.01
Duration of operation, hours	241 (180;290)	315 (270;360)	0.046
Duration of inotropic support, hours	38 (10;60)	36 (24;72)	0.4
Duration of mechanical ventilation, hours	15,5 (4;43)	24(24;36)	0.04
Duration of treatment in the ICU, days	3 (2;5)	2 (2;3)	0.08
LVEF, %	70,5 (61;75)	68 (65;73)	0.8
RVOT peak gradient	16 (9;24)	16 (11;19)	0.8
Complications, n (%)	2 (10%)	3 (14%)	0.9
Duration of hospitalization, days	15 (12;20)	16 (15;22)	0.2

ICU=intensive care unit; LVEF=left ventricular ejection fraction; mBTs=modified Blalock-Taussig shunt; RVOT=right ventricular outflow tract

## DISCUSSION

RVOT stenting is a potentially viable alternative to the creation of an mBTs, offering advantages such as antegrade pulsatile flow of venous blood into the pulmonary bed with high oxygen saturation of arterial blood and rapid stabilization of hemodynamics and organ perfusion due to the absence of a decrease in diastolic arterial pressure^[9,18-21]^.

Within the scope of our prospective, two-center randomized study, a comparative evaluation of the outcomes of two palliative treatment methods - stenting of the RVOT and creation of an mBTs - was conducted. The study aimed to investigate their impact on pulmonary flow development, describe major cardiovascular events in the interstage period, and assess the immediate outcomes of complete correction.

A number of studies confirm that stenting of the RVOT in critical forms of ToF is an effective and safe procedure, with a mortality rate of 0% (Sandoval J. P. et al.^[22]^) and 1.7% (Quandt D, et al.^[15]^) in the early postoperative period. The results of delayed surgical treatment after stenting of the RVOT are comparable to the outcomes of primary complete correction in older age groups. In our study, the mortality rate in the early postoperative period after palliative treatment was also 0%. Only one lethality outcome (4.8%) was observed in the interstage period in the stent group, which was associated with non-cardiac pathology. In other aspects, no major cardiovascular events were identified in the study groups during the interstage period. However, literature describes cases of persistent desaturation after RVOT stenting, caused by the development of obstruction in the proximal part of the RVOT, which can deform the corresponding section of the stent. In such cases, balloon angioplasty or the placement of another stent with coverage of the previous one has been carried out to address the resulting obstruction in the proximal part of the RVOT^[15,22-24]^. Several authors have reported cases of persistent desaturation after RVOT stenting, necessitating additional creation of an mBTs^[15,22]^. In turn, after the creation of mBTs, cases of thrombosis have been observed, requiring reformation of the mBTs or stenting of the pathway to the PA^[15,25]^. In a study assessing the impact of both palliative treatment methods on PA growth, it was shown that stenting of the RVOT leads to faster, significant, and more uniform development of the PA compared to the creation of an mBTs. Over the observation period, the stent group showed a significant increase in the right PA (∆*z* score) of 2.2, which was significantly higher than the mBTs group with a score of 1.1. A similar pattern was observed in the left PA, with a ∆*z* score of 2.6 in the stent group, significantly exceeding the score of 1.6 in the mBTs group. Using a mixed-effects model, it was also found that the rate of growth of the right and left PAs was significantly higher in the stent group. In retrospective studies by several authors^[15,23,24,26]^, similar results have been reported, confirming faster and more uniform growth of the branches of the PA after stenting of the RVOT. In studies conducted by a group of authors from Toronto, it was described that in 80% of patients at the time of stenting of RVOT the Nakata index was < 100 mm^2^/m^2^, while at the time of complete correction, only 16% of children had a Nakata index remaining < 100 mm^2^/m^2^[^22]^. In the study by Dohlen et al.^[23]^, an increase in the Nakata index from a mean of 56 mm^2^/m^2^ (range 21-77) to 150 mm^2^/m^2^ (range 123-231) was described. In our study, the stent group showed a ∆ (increase) in the Nakata index of 105.2 mm^2^/m^2^, which was significantly higher than the mBTs group with a ∆ of 58.2 mm^2^/m^2^. Some authors use the difference in Nakata index as a measure for evaluating the growth of the PA. In our analysis, we also used the difference in the Nakata index^[2]^ to estimate the PA growth rate. The mean index of growth (mm^2^/m^2^/month), representing the speed of growth of the PA, was 35.9 mm^2^/m^2^/month in the RVOT stent group and 22.1 mm^2^/m^2^/month in the group of mBTs.

Binary logistic regression analysis revealed that stenting increased the odds of achieving a Nakata index ≥ 200 mm^2^/m^2^ by 7.78 times compared to the formation of a modified mBTs. Another important consideration is the fact that when assessing the growth of the PAs using the Nakata and the *z* score indices, the interventions on the PAs themselves during surgical procedures could potentially alter the data used to estimate the growth rate. This is why it is likely that the use of the lower lobe index in these patients for assessing PA growth is more reliable since the lower lobe branches of the PAs remain intact.

Another interesting hypothesis is that during stenting of the RVOT, the development of the PA improves due to the balanced growth of both the right and left PAs. This is because the PAs themselves are not directly affected during the procedure. Daniel Quandt et al. demonstrated in their study that patients who underwent initial RVOT stenting exhibited improved and more consistent growth of the branch PAs compared to those who underwent an mBTs^[15]^. Indeed, our study partially addressed this issue, although it was not the primary endpoint. We were able to assess this by measuring the lower lobe index, as the lower branch remains intact from the surgical intervention in mBTs procedures. However large multicenter studies needed to validate this hypothesis.

One of the important questions is the reconstruction of the RVOT during delayed complete correction. Colleagues from Birmingham describe in a single-center retrospective study that the type of reconstruction of the RVOT after stenting of the pulmonary valve or creation of an mBTs was similar^[15]^. In 58% of cases after pulmonary valve stenting or creation of an mBTs, transannular patch repair of the RVOT was performed. In 30% after stenting and 31% after mBTs creation, PA prosthetic replacement was done. It was also noted that the number of plastic interventions on the PA branches after pulmonary valve stenting was lower (15%) compared to the results after forming the mBTs (31%).

According to the results of our study, the type of reconstruction of the outflow tract to the PA did not statistically differ between the groups. Transannular patch repair of the RVOT was performed in 12 (60%) cases out of 20 in the stent group and in 15 (71.4%) out of 21 cases in the mBTs group. Eight children (40%) in the stent group and six (28.6%) in the mBTs group underwent prosthetic reconstruction of the outflow tract to the PA. The choice of reconstruction method was determined by the assessment of the risk of right ventricular insufficiency. All patients in the mBTs group required additional plastic intervention on the stenosed PA after mBTs formation. In one case in the stent group, after prosthetic replacement of the PA, stenoses of the branch PAs were detected, requiring additional stenting.

In the intergroup comparison, the duration of the complete correction surgery in the stent group was significantly shorter. The duration of the complete correction procedure in patients from the stent group was considered to be significantly shorter primarily due to factors such as the absence of adhesion formation and a reduced occurrence of intraoperative complications. However, the duration of aortic occlusion and artificial circulation was significantly higher in the pulmonary valve stenting group, which can be explained by the additional time required for explantation of the stent from the RVOT.

Furthermore, it should also be emphasized that despite the positive results associated with RVOT stenting, there are still potential complications to be aware of, including perforation of the distal PA, different forms of PA injury, migration or fracture of the stent, arrhythmias, coronary artery compression, and the development of postoperative infectious complications. After acquiring proficiency and overcoming the learning curve, it is expected that there will be a reduction in the occurrence of complications.

### Limitations

Considering the very low percentage of children with severe forms of tetralogy of Fallot, the sample size in a prospective randomized study is limited. This may be insufficient to determine the presence or absence of differences in certain studied parameters and may limit the significance of the obtained results.

## CONCLUSION

Stenting of the RVOT and creating an mBTs both reduce the severity of PA hypoplasia, as assessed by the Nakata index. However, in intergroup comparison, the increase in the Nakata index after stenting is more significant. Therefore, both stenting of the RVOT and mBTs are important tools in the arsenal of pediatric cardiologists and surgeons. RVOT stenting provides hemodynamic stabilization and symmetric growth of the PA compared to mBTs.

## Figures and Tables

**Table 4:** Binary logistic regression results.

Variable	Univariate analysis		Multivariate analysis	
	OR (95% CI)	*P*-value	OR (95% CI)	*P*-value
Age	0.986 (0,964; 1,007)	0.212	1,017 (0,983; 1,059)	0.354
Prematurity	0,846 (0,107; 4,987)	0.858	0,200 (0,007; 3,066)	0.288
Group	9.000 (2,180; 48,321)	0.004	7,777 (1,612; 49,924)	0.016
Duration of the interstage period	0,999 (0,991; 1,004)	0.641	0,998 (0,990; 1,003)	0.429

CI=confidence interval; OR=odds ratio

## References

[1] Al Habib HF, Jacobs JP, Mavroudis C, Tchervenkov CI, O'Brien SM, Mohammadi S (2010). Contemporary patterns of management of tetralogy of fallot: data
from the society of thoracic surgeons database. Ann Thorac Surg.

[2] Petrucci O, O'Brien SM, Jacobs ML, Jacobs JP, Manning PB, Eghtesady P. (2011). Risk factors for mortality and morbidity after the neonatal
blalock-taussig shunt procedure. Ann Thorac Surg.

[3] Godart F, Qureshi SA, Simha A, Deverall PB, Anderson DR, Baker EJ (1998). Effects of modified and classic blalock-taussig shunts on the
pulmonary arterial tree. Ann Thorac Surg.

[4] Jahangiri M, Lincoln C, Shinebourne EA. (1999). Does the modified blalock-taussig shunt cause growth of the
contralateral pulmonary artery?. Ann Thorac Surg.

[5] Kulkarni H, Rajani R, Dalvi B, Gupta KG, Vora A, Kelkar P. (1995). Effect of blalock taussig shunt on clinical parameters, left
ventricular function and pulmonary arteries. J Postgrad Med.

[6] Caspi J, Pettitt TW, Mulder T, Stopa A. (2008). Development of the pulmonary arteries after the norwood
procedure: comparison between blalock-taussig shunt and right
ventricular-pulmonary artery conduit. Ann Thorac Surg.

[7] Pruetz JD, Badran S, Dorey F, Starnes VA, Lewis AB. (2009). Differential branch pulmonary artery growth after the norwood
procedure with right ventricle-pulmonary artery conduit versus modified
blalock-taussig shunt in hypoplastic left heart syndrome. J Thorac Cardiovasc Surg.

[8] Younis Memon MK, Akhtar S, Mohsin M, Ahmad W, Arshad A, Ahmed MA. (2019). Short and midterm outcome of fallot's tetralogy repair in
infancy: a single center experience in a developing country. J Ayub Med Coll Abbottabad.

[9] Linnane N, Nasef MA, McMahon CJ, McGuinness J, McCrossan B, Oslizlok P (2021). Right ventricular outflow tract stenting in symptomatic infants
without the use of a long delivery sheath. Catheter Cardiovasc Interv.

[10] Van Arsdell GS, Maharaj GS, Tom J, Rao VK, Coles JG, Freedom RM (2000). What is the optimal age for repair of tetralogy of
fallot?. Circulation.

[11] Fiore AC, Tobin C, Jureidini S, Rahimi M, Kim ES, Schowengerdt K. (2011). A comparison of the modified blalock-taussig shunt with the right
ventricle-to-pulmonary artery conduit. Ann Thorac Surg.

[12] Jo TK, Suh HR, Choi BG, Kwon JE, Jung H, Lee YO (2018). Outcome of neonatal palliative procedure for pulmonary atresia
with ventricular septal defect or tetralogy of fallot with severe pulmonary
stenosis: experience in a single tertiary center. Korean J Pediatr.

[13] Zheng S, Yang K, Li K, Li S. (2014). Establishment of right ventricle-pulmonary artery continuity as
the first-stage palliation in older infants with pulmonary atresia with
ventricular septal defect may be preferable to use of an arterial
shunt. Interact Cardiovasc Thorac Surg.

[14] Lenoir M, Pontailler M, Gaudin R, Gerelli S, Tamisier D, Bonnet D (2017). Outcomes of palliative right ventricle to pulmonary artery
connection for pulmonary atresia with ventricular septal
defect. Eur J Cardiothorac Surg.

[15] Quandt D, Ramchandani B, Penford G, Stickley J, Bhole V, Mehta C (2017). Right ventricular outflow tract stent versus BT shunt palliation
in tetralogy of fallot. Heart.

[16] Chubb H, Simpson JM. (2012). The use of Z-scores in paediatric cardiology. Ann Pediatr Cardiol.

[17] Di Donato RM, Jonas RA, Lang P, Rome JJ, Mayer JE Jr (1991). Castaneda AR. Neonatal repair of tetralogy of fallot with and
without pulmonary atresia. J Thorac Cardiovasc Surg.

[18] Barron DJ. (2013). Tetralogy of fallot: controversies in early
management. World J Pediatr Congenit Heart Surg.

[19] Quandt D, Ramchandani B, Stickley J, Mehta C, Bhole V, Barron DJ (2017). Stenting of the right ventricular outflow tract promotes better
pulmonary arterial growth compared with modified blalock-taussig shunt
palliation in tetralogy of fallot-type lesions. JACC Cardiovasc Interv.

[20] Dryzek P, Mazurek-Kula A, Moszura T, Sysa A. (2008). Right ventricle outflow tract stenting as a method of palliative
treatment of severe tetralogy of Fallot. Cardiol J.

[21] Cools B, Boshoff D, Heying R, Rega F, Meyns B, Gewillig M. (2013). Transventricular balloon dilation and stenting of the RVOT in
small infants with tetralogy of fallot with pulmonary
atresia. Catheter Cardiovasc Interv.

[22] Sandoval JP, Chaturvedi RR, Benson L, Morgan G, Van Arsdell G, Honjo O (2016). Right ventricular outflow tract stenting in tetralogy of fallot
infants with risk factors for early primary repair. Circ Cardiovasc Interv.

[23] Dohlen G, Chaturvedi RR, Benson LN, Ozawa A, Van Arsdell GS, Fruitman DS (2009). Stenting of the right ventricular outflow tract in the
symptomatic infant with tetralogy of fallot. Heart.

[24] Stumper O, Ramchandani B, Noonan P, Mehta C, Bhole V, Reinhardt Z (2013). Stenting of the right ventricular outflow tract. Heart.

[25] Barron DJ, Jegatheeswaran A. (2021). How and when should tetralogy of fallot be palliated prior to
complete repair?. Semin Thorac Cardiovasc Surg Pediatr Card Surg Annu.

[26] Marshall AC, Love BA, Lang P, Jonas RA, del Nido PJ, Mayer JE (2003). Staged repair of tetralogy of fallot and diminutive pulmonary
arteries with a fenestrated ventricular septal defect patch. J Thorac Cardiovasc Surg.

